# Proteome Profiling of Exosomes Purified from a Small Amount of Human Serum: The Problem of Co-Purified Serum Components

**DOI:** 10.3390/proteomes7020018

**Published:** 2019-04-28

**Authors:** Mateusz Smolarz, Monika Pietrowska, Natalia Matysiak, Łukasz Mielańczyk, Piotr Widłak

**Affiliations:** 1 Maria Skłodowska-Curie Institute—Oncology Center, Gliwice Branch, 44-101 Gliwice, Poland; mateusz.smolarz@io.gliwice.pl (M.S.); monika.pietrowska@io.gliwice.pl (M.P.); 2 Department of Histology and Cell Pathology, School of Medicine with Division of Dentistry in Zabrze, Medical University of Silesia, 41-800 Zabrze, Poland; nmatysiak@sum.edu.pl (N.M.); lmielanczyk@sum.edu.pl (Ł.M.)

**Keywords:** exosome, mass spectrometry, serum proteome, size-exclusion chromatography

## Abstract

Untargeted proteomics analysis of extracellular vesicles (EVs) isolated from human serum or plasma remains a technical challenge due to the contamination of these vesicles with lipoproteins and other abundant serum components. Here we aimed to test a simple method of EV isolation from a small amount of human serum (<1 mL) using the size-exclusion chromatography (SEC) standalone for the discovery of vesicle-specific proteins by the untargeted LC–MS/MS shotgun approach. We selected the SEC fraction containing vesicles with the size of about 100 nm and enriched with exosome markers CD63 and CD81 (but not CD9 and TSG101) and analyzed it in a parallel to the subsequent SEC fraction enriched in the lipoprotein vesicles. In general, there were 267 proteins identified by LC–MS/MS in exosome-containing fraction (after exclusion of immunoglobulins), yet 94 of them might be considered as serum proteins. Hence, 173 exosome-related proteins were analyzed, including 92 proteins absent in lipoprotein-enriched fraction. In this set of exosome-related proteins, there were 45 species associated with the GO cellular compartment term “extracellular exosome”. Moreover, there were 31 proteins associated with different immune-related functions in this set, which putatively reflected the major role of exosomes released by immune cells present in the blood. We concluded that identified set of proteins included a bona fide exosomes components, yet the coverage of exosome proteome was low due to co-purified high abundant serum proteins. Nevertheless, the approach proposed in current work outperformed other comparable protocols regarding untargeted identification of exosome proteins and could be recommended for pilot exploratory studies when a small amount of a serum/plasma specimen is available.

## 1. Introduction

Extracellular vesicles (EVs) have gained a large interest in the recent decade due to their role in cell-to-cell communication. Exosomes are the smallest virus-sized EVs (30–150 nm). They derive from the inward budding of the endosomal membrane to form the multivesicular body that fuses with the plasma membrane to release exosomes to the extracellular space [1]. The exosomal cargo consists of precisely selected molecules located inside these vesicles or associated with their membrane [2]. Exosomes could reach recipient cells in the local environment (paracrine mode) or could be transported to distant tissues via the circulation system (endocrine mode). Numerous investigations revealed an important role of exosomes in intercellular communication under both normal and pathological conditions [3,4]. Studies on exosomes have been focused on their role in cancer, as a potential source of tumor biomarkers (miRNA, tumor-specific proteins) [5,6,7,8] and as valuable mediators of a cross-talk between tumor and immune system cells [4,9]. To study the function of exosomes it is essential to know their molecular content, which specifically reflects the phenotype of parent cells. Currently, proteins, miRNAs, mRNAs, and metabolites (organic acids and their derivatives, nucleotides, sugars and derivatives, carnitines, vitamin B/related metabolites and amines) were already identified in exosomes purified from different cells types and biological fluids [10,11,12]. Thousands of different macromolecules were reported to be present in exosomes, which could be found in EV-oriented knowledge-bases like Exocarta (www.exocarta.org) or Vesiclepedia (www.microvesicles.org).

Exosomes and their components can be investigated for diagnostic purposes as they are secreted into the bloodstream. In general, peripheral blood is an easily accessible material for diagnostics and important clinical information on disease stage and response to treatment can be achieved using this biospecimen. Both serum and plasma contain exosomes and could be used for their purification. However, it is important to note that at the same time these specimens contain circulating cells and sub-cellular structures like cell debris and other classes extracellular vesicles exemplified by microvesicles (otherwise termed ectosomes) and apoptotic bodies. Therefore the content of these circulating structures together with “soluble” components of blood (e.g., lipoproteins and abundant serum proteins) should be treated as a background “contamination” in term of studies oriented on exosomes. Moreover, the population of exosomes present in the blood is very heterogenous because circulating vesicles are released by different types of cells, either circulating blood cells or cells having contact with circulation (e.g., endothelial cells). The majority of circulating exosomes are released by platelets, lymphocytes, dendritic cell, and other immune cells; it is estimated that exosomes released by these cells comprise 80–90% of serum/plasma exosomes [13,14,15]. Moreover, a large fraction of circulating exosomes are released by endothelial cells and, in the blood of cancer patients, by malignant cells (these exosomes are termed tumor-derived exosomes, TEXs) [14,16]. Discrimination of different populations of exosomes is possible based on their actual surface antigens. Therefore, the identification and isolation of TEXs circulating in the blood, which are very interesting diagnostic targets, represents a challenging issue requiring knowledge of specific tumor markers [16].

Multiple techniques have been used to isolate exosomes from a cell culture media and from different biofluids such as serum or plasma [17,18,19,20]. The most popular approaches involve ultracentrifugation or precipitation with poly-ethylene glycol-like substances, which are based on sedimentation/density and physicochemical properties of these vesicles, respectively. Currently, replacement or complementation of these methods by size-exclusion chromatography (SEC) was proposed [17,21,22,23,24,25], which enables separation of vesicles based on their actual size. Two major fractions of small vesicles, having average sizes of 100 and 30 nm, are frequently detected in preparation of exosomes isolated from different sources. Several lines of evidence indicate that these smaller vesicles represent lipoproteins [25,26,27]. However, it is important to note, that these two fractions of vesicles are observed not only in material isolated from biofluids like plasma/serum but also from cell culture media [28]. Nevertheless, these two fractions of vesicles are frequently analyzed as a mixture because they are not separated by precipitation, currently the simplest and most popular approach in exosome studies [28]. However, the presence of lipid vesicles (lipoproteins and chylomicrons) could be a serious obstacle in many types of exosome studies. Moreover, serum and plasma are very difficult substrates for any proteomics study because the presence of albumin and other abundant “soluble” proteins impairs the ability to detected low-abundant proteins by untargeted shot-gun approaches [29]. Hence, the combination of ultracentrifugation/density cushion and size-exclusion chromatography was recently proposed for separation of exosomes from lipid vesicles and other EVs present in human plasma and other biofluids [25,26]. However, such combined procedures are more laborious and require a large amount of biomaterial, which could be a disadvantage in routine applications. Here we aimed to characterize the feasibility of shotgun proteomics profiling of exosomes purified from a small amount of serum (<1mL) by the micro-SEC approach successfully implemented in a proteomics study of exosomes released in vitro to cell culture media [30].

## 2. Materials and Methods

### 2.1. Isolation of Extracellular Vesicles from Human Serum

The serum used for the study was isolated from the blood of four healthy volunteers who provided written informed consent (permission of the local Bioethical Committee no. KB/430-16/13). Five mL of blood was collected into an anticoagulant-free tube (Becton Dickinson, Franklin Lakes, NJ, USA; 367955), incubated for 30 min at 20 °C then centrifuged at 1000 × *g* for 10 min at 4 °C. The supernatant (i.e., serum) was transferred to clean tubes and stored at −80 °C until use. Small extracellular vesicles were isolated by micro-SEC (size exclusion chromatography) from 0.5 mL of serum. Serum was pre-purified by a series of centrifugations at 1,000 and 10,000 × *g* for 10 and 30 min at 4 °C, respectively, then the supernatant was filtrated using a 0.22 µm syringe filter unit (Roth, Karlsruhe Germany; PA49.1). Filtered serum was loaded onto an Econo-Pac 10DG column (BioRad, Hercules, CA, USA; 732-2010) filled with 10 mL of Sepharose CL-2B (GE Healthcare, Chicago, USA; 17014001) at 6 cm length. The first 0.5 mL fraction was collected right after the sample had been loaded (void volume) then subsequent fractions (0.5 mL each) were eluted using PBS. The presence of EVs in the collected fractions was detected by Western blot using typical exosome markers (CD9, CD63, CD81, and TSG101 [2,17,18,19]); EVs started to be eluted in fraction 7.

### 2.2. Western Blot Analysis

The concentration of proteins in the analyzed samples was assessed using the PierceTM BCA Protein Assay kit (Thermo Fisher Scientific, Waltham, MA, USA; 23225) according to the manufacturer’s instructions. Ten µL of SEC fractions or amounts corresponding to 0.2 µg of proteins were mixed with loading buffer to a final concentration of 2% (*v*/*v*) SDS and optionally 100 mM DTT, then denatured for 5 min at 95 °C. Samples were separated by 12% or 5% SDS-polyacrylamide gel electrophoresis followed by wet electro-transfer onto nitrocellulose membranes (Thermo Fisher Scientific, Waltham, MA, USA; 88018). Membranes were blocked for 1 h in 5% non-fatty milk and 0.1% Tween in PBS, and then primary antibody (anti-CD63: Invitrogen, 10628D, 1:1500; anti-CD9: Santa Cruz Biotechnology, sc-13118, 1:500; anti-CD81: Biorbyt, orb388959, 1:500; anti-TSG101: Becton Dickinson, 612697, 1:800; anti-CD35: Abcam, ab133293, 1:1000; anti-ApoB: Santa Cruz Biotechnology, sc-393636, 1:200; anti-PHB1: Cell Signaling Technology, 2426S, 1:1000; anti-Grp94: Cell Signaling Technology, 2104S, 1:1000) was added for overnight incubation at 4 °C. After triplicate washes, secondary antibody conjugated with HRP was added for 1 h at room temperature. Detection of the bands by chemiluminescence was carried out using WesternBright Sirius HRP substrate (Advansta, San Jose, CA, USA, K-12043-D10) according to the manufacturer’s instructions. To analyze CD63 and CD81 SDS-PAGE was performed under non-reducing conditions.

### 2.3. Characterization of Extracellular Vesicles

The size distribution profile of EVs was estimated by the dynamic light scattering (DLS) measurement using a Zetasizer Nano-ZS90 instrument (Malvern Instruments, Malvern, UK). Fifty μL of SEC fractions were analyzed at 20 °C immediately after isolation in disposable low-volume cuvettes (ZEN0118, Malvern Instruments, Malvern, UK). The dispersant refractive index was 1.330 (ICN PBS Tablets) and equilibration time was set for 30 s. The results were the average of 5 measurements consisting of 10 runs (Malvern Zetasizer Software 7.12; Malvern Instruments, Malvern, UK was used for data analysis).

### 2.4. Transmission Electron Microscopy (TEM)

To assess the size and morphology of extracellular vesicles using transmission electron microscopy, SEC samples were concentrated from 500 μL to 25 μL using Vivaspin500 ultrafiltration tubes (Sartorius, Göttingen, Germany, VS0102) according to the manufacturer’s instructions. Then, equal volumes of the EV sample and 4% paraformaldehyde were mixed and incubated at 4° C until analysis. Exosomes fixed in 4% PFA were visualized using transmission electron microscopy according to Thery et al. [31]. Briefly, 5 µL of exosome suspension was deposited onto carbon-coated copper EM grids and left for 20 min in a dry environment. A 100 µL drop of PBS was put on a sheet of Parafilm and grids with exosomes were transferred on the buffer droplet for 2 min. Next, the grids were transferred to a 50 µL drop of a 1% glutaraldehyde (Polysciences Inc., Warrington, PA, USA, 01909-100) for 5 min followed by transferring to a 100 µL drop distilled water for 2 min. Step with distilled water was repeated seven times, and then samples were contrasted on a 50 µL drop of uranyl-oxalate solution (pH 7) for 5 min and transferred to a 100 µL drop of methyl-cellulose-UA for 10 min on ice. The methyl-cellulose-UA mixture was prepared by mixing a 4% uranyl acetate (Polyscience Inc., Warrington, USA, 21447) solution and 2% methyl-cellulose in a ratio 100 µL/900 µL, respectively. The grids were removed from the last droplets using stainless steel loops and excess fluid was removed. For immunogold labeling analysis, the adsorbed exosomes on the carbon-formvar coated nickel EM grids were floated sequentially on droplets of PBS buffer (for 3 min), afterward on droplets of 50 mM ammonium chloride (5 min), and blocking solution (1% BSA in PBS, for 10 min). Then, grids were transferred on primary antibody droplets diluted 1:20 for 60 min (anti-CD9, anti-CD63, or anti-ApoB), before transferring to a 0.1% BSA (5 times 2 min), secondary antibody Protein A Gold 10 nm diluted 1:50, for 60 min (BBInternational, Cardiff, UK, Batch 15249), and finally PBS buffer droplets (5 times 2 min). Further, grids were fixed by 1% glutaraldehyde for 5 min and followed by rinsing in distilled water (5 times 2 min), contrasted and embedded in UA-methyl-cellulose as above. The grids with exosomes embedded in methyl-cellulose-UA were air-dried and the samples were visualized using FEI Tecnai Spirit G2 BioTWIN at 120 kV acceleration.

### 2.5. Sample Preparation for LC–MS/MS

SEC samples were concentrated using Vivaspin500 ultrafiltration tubes as described above, then 10 μL of the concentrate (corresponding to 0.5–2 µg of proteins) was mixed with 10 μL of 50 mM NH_4_HCO_3_ and 1.5 μL of 0.1 M DTT, and then incubated at 95 °C for 5 min. The samples were allowed to cool, then mixed with 3 μL of 0.1M iodoacetamide and incubated 20 min at room temperature in the dark. Proteins were digested with trypsin (Promega, Madison, WI, USA, V5111) using 0.1 μg trypsin per 1 μg proteins for 18 h at 37 °C, then trifluoroacetic acid (TFA) was added to a final concentration 0.1%. If necessary, the reaction was performed in replicates combined afterward. Tryptic peptides were purified using a C18 StageTips prepared by stacking 6 layers of Empore™ Octadecyl C18 extraction disk (3M, Maplewood, MN, USA) in a 0.2 mL pipette tip. The C18 spin-column was preconditioned with 100% methanol followed by 60% acetonitrile (ACN) with 0.1% TFA and washed with 0.1% TFA. Peptides bound to the column were washed with 5% methanol with 0.1% TFA (3 times) and 0.1% TFA (2 times), then eluted with 60% ACN with 0.1% TFA; washes and elution by centrifugation at 4000 RCF for 5 min. Eluates were evaporated to dryness in a vacuum centrifuge, peptides were reconstituted in 20 µL of LC–MS grade water and subjected to peptide assay using tryptophan fluorescence method [32]. After the measurement samples were acidified with TFA to achieve the final concentration of 0.1% (*v*/*v*) and subjected to LC–MS/MS analysis.

### 2.6. Protein Identification by LC-MS/MS

Tryptic peptides (approx. 0.9 μg) were separated on a reverse phase Acclaim PepMap RSLC nanoViper C18 column (75 µm × 25 cm, 2 µm granulation (Thermo Fisher Scientific, Waltham, MA, USA) using acetonitrile gradient (from 4 to 60%, in 0.1% formic acid) at 30 °C and a flow rate of 300 nL/min (for 180 min) by Ultimate 3000 Nano system (Thermo Fisher Scientific, Waltham, MA, USA) and analyzed on-line using a Q Exactive Plus mass spectrometer (Thermo Fisher Scientific, Waltham, MA, USA). The spectrometer was operated in data-dependent MS/MS mode with survey scans acquired at a resolution of 70,000 at *m*/*z* 200 in MS mode, and 17,500 at *m*/*z* 200 in MS2 mode. Spectra were recorded in the scanning range of 300–2000 *m*/*z* in the positive ion mode. Higher energy collisional dissociation (HCD) ion fragmentation was performed with normalized collision energies set to 25. All raw data obtained for each dataset were imported into Protein Discoverer v.1.4 (Thermo Fisher Scientific) < Thermo raw files > for protein identification and quantification, then Mascot and Sequest engines were used for database searches. Protein identification was performed using reviewed Swiss-Prot human database with a precision tolerance 10 ppm for peptide masses and 0.08 Da for fragment ion masses. Protein was considered as positively identified if a peptide score of specific peptide reached the significance threshold FDR = 0.01 (assessed by the Percolator algorithm), a peptide had at least 6 amino acid sequences and was classified as unique by at least one search engine (Mascot or Sequest).

### 2.7. Bioinformatics Analysis

A list of genes corresponding to identified proteins was annotated at Gene Ontology (GO) using g:profiler web server (http://biit.cs.ut.ee/gprofiler/) [33]. A list of genes corresponding to all identified proteins was used as the reference set, and then statistical over-representation of GO terms associated with genes present in a given subset was estimated based on the hypergeometrical distribution. To allow direct pairwise comparison of two subsets of proteins an original method based on clustering of GO term was used [34]. In order to define groups of functionally related GO terms, each protein/gene was annotated by its corresponding GO terms, taking into account the hierarchical structure of a Gene Ontology graph; only GO terms to which at least two genes were annotated and showing statistically significant over-representation (*p* < 0.05) were selected for further analyses. Then, groups (clusters) of similar GO terms were identified by applying a hierarchical clustering method with G-SESAME similarity measure [35] used to compute the distance matrix T; the number of clusters was arbitrary set to 20 for biological processes (BP) and 10 for molecular functions (MF) and cellular compartment (CC). For each cluster, a list of genes that were annotated to GO terms from that cluster was obtained, and a contribution of genes present in a cluster was computed as a percentage of all genes detected in a subset. Finally, the significance of differences between subsets regarding gene contribution to clusters of GO terms was estimated using the Fisher exact test.

## 3. Results

**Figure 1:**
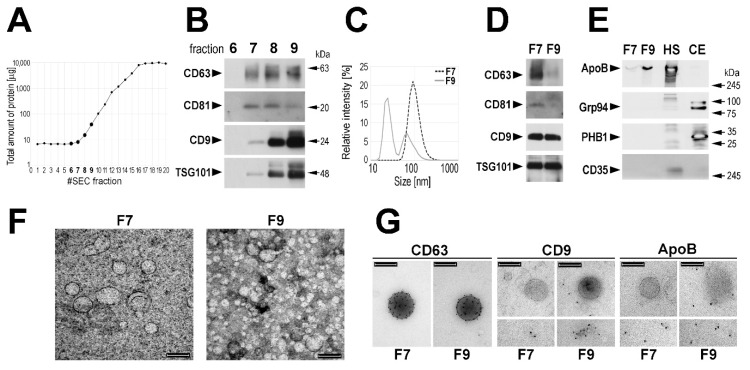
Characteristics of vesicles purified from human serum. Amount of total serum proteins (**A**) and exosome markers (**B**) in the SEC fractions; selected proteins were analyzed by Western blot in the same volume (10 µl) of each fraction. (**C**) Size of vesicles measured by DLS in fractions F7 and F9. (**D**,**E**) Level of selected proteins analyzed by Western blot in fractions F7 and F9 using 0.2 µg of proteins in both fractions; HS—human serum and CE—HCT116 cell extract used as positive controls (arrows represent positions of protein molecular weight markers). (**F**) Imaging of vesicles detected in fractions F7 and F9 by the TEM. (**G**) The occurrence of CD63, CD9, and ApoB in vesicles present in fractions F7 and F9 analyzed by the immunogold-EM (smaller inserts illustrate the character of CD9- and ApoB-positive structures); the scale bars represent 100 nm.

Circulating EVs were isolated from 0.5 mL of serum using the size-exclusion chromatography (SEC) approach. Total protein amount and levels of specific proteins considered as exosome markers (CD63, CD81, CD9, and TSG101 [2,17,18,19]) were monitored in the subsequent SEC fractions. The majority of serum proteins started to be eluted in fraction 10 with the maximum between fractions 16 and 20 (Figure 1A). On the other hand, “exosome markers” were detected by Western blotting starting from fraction 7 (Figure 1B). Noteworthy, very low amount of total protein was measured in this fraction, which increased in subsequent fractions: ca. 3–5-fold in fraction 8 and 10–20-fold in fraction 9. The average size of the major type of vesicles measured in fraction 7 (F7) by the dynamic light scattering (DLS) was approx. 95 nm, while the average size of the major type of vesicles detected in fraction 9 (F9) was approx. 25 nm (Figure 1C); fraction 8 represented a mixture of both types of vesicles. The size of vesicles present in the SEC fractions was compared in preparations obtained from sera of four healthy donors, and we found that the average size of the major type of vesicles detected in fractions F7 was between 50 and 100 nm, while the average size of the major type of vesicles detected in fractions F9 was between 20 and 25 nm (Supplementary Figure S1A). Hence, we focused afterward on fraction F7 and used fraction F9 as a reference. Then we compared these two fractions regarding the actual levels of proteins considered to be “exosome markers”. We found that fractions F7 (enriched in “larger” vesicles) showed higher relative content of CD63 and CD81 when compared to fractions F9. On the other hand, CD9 and TSG101 were not enriched in fraction F7 (Figure 1D). Moreover, the higher relative content of CD63 and CD81 but not of CD9 and TSG101 in fraction F7 than in fraction F9 (enriched in “smaller” vesicles) was confirmed when corresponding fractions from sera of four healthy donors were analyzed (Supplementary Figure S1B,C).

Moreover, the level of other proteins was compared by Western blotting in fractions F7 and F9 (representative data are shown in Figure 1E). We found that ApoB, the primary component of LDL and VLDL lipoproteins could be readily detected in fraction F9, yet a small amount of this protein was detected also in fraction F7. On the other hand, Grp94 and PHB1, characteristic for endoplasmic reticulum and mitochondria, respectively, were detected neither in fraction F7 nor in fraction F9. Moreover, complement receptor 1 (CD35/CR1), a protein reported to be present specifically in microvesicles (also known as ectosomes) [36], was detected neither in fraction F7 nor in fraction F9. Therefore, we concluded that neither cell debris nor larger EVs (i.e., microvesicles/ectosomes) were present in analyzed fractions, yet VLDL/LDL lipoproteins should be expected in fraction F9.

Vesicles present in fractions F7 and F9 were further characterized using a transmission electron microscopy (TEM) and immunogold-EM. Vesicles detected in the fraction F7 showed sizes in the 80–100 nm range. On the other hand, the majority of vesicles detected in the fraction F9 showed sizes around and below 30 nm, yet a small number of larger vesicles (approx. 100 nm) could be also detected (Figure 1F). Only larger vesicles (approx. 100 nm) detected in both F7 and F9 were labeled with the anti-CD63 antibody. On the other hand, these larger vesicles were not labeled with the anti-CD9 antibody in either F7 or F9, yet some labeling of smaller vesicles (≤30 mn) could be detected in F9. Furthermore, larger vesicles were not stained with the anti-ApoB antibody in either F7 or F9, and only smaller vesicles were ApoB-positive (Figure 1G). In conclusion, though a specific way of the vesicle biogenesis via multivesicular bodies was not verified in the current study, the EVs present in fraction F7 could be called “exosomes” for simplicity.

**Figure 2:**
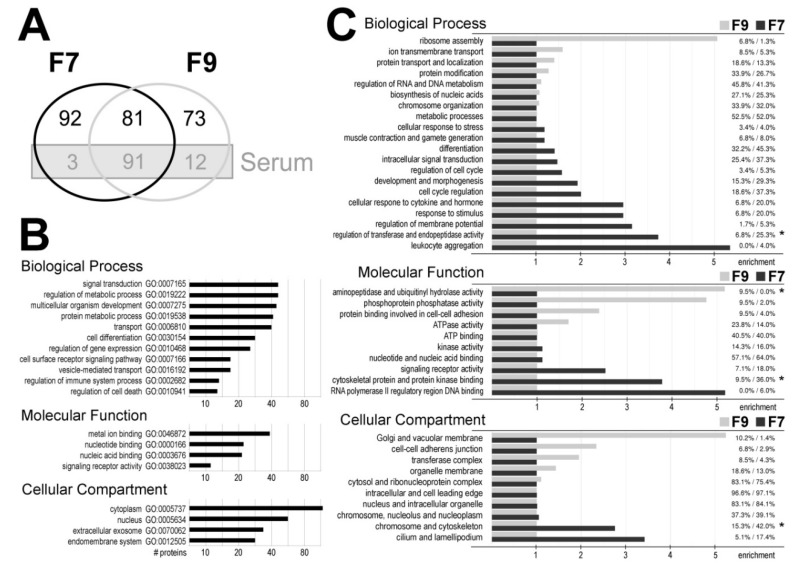
Proteomics profiling of vesicles purified from human serum. (**A**) Numbers of proteins identified in fractions F7 and F9; marked is a subset of “background” proteins detected in unfractionated serum. (**B**) GO terms associated with the most numerous groups of proteins detected in fraction F7 (173 proteins, background serum proteins excluded); showed are numbers of associated proteins. (**C**) Clusters of GO terms associated with proteins specific for F7 (92 proteins) and F9 (73 proteins); showed is a percentage of proteins associated with a cluster (F9 vs. F7) and relative enrichment of each protein subset in a given cluster represented by horizontal bars (asterisks marked statistically significant differences; *p* < 0.05).

Proteins present in fractions F7 and F9 were identified using the untargeted shotgun LC–MS/MS approach. Technical replicates of the SEC purification were combined and material corresponding to 1 mL of serum from one individual was analyzed for fraction F7 along with a comparable amount of proteins from fraction F9. In general, proteins encoded by the 352 unique genes were identified and analyzed (detected immunoglobulins were excluded from the analysis); these proteins are listed in the Supplementary Material Table S1. It is noteworthy that the major serum proteins typically detected in the unfractionated human serum using the same shotgun approach [37] represented a large subset (106 species) of proteins identified in fractions F7 and F9; such serum proteins comprised of more than 50% of proteins common for both fraction (including 14 different apolipoproteins). These major serum proteins were considered as “contaminants” and removed from further analysis. Therefore we assumed that 173 proteins were characteristic for vesicles present in fraction F7 (including 92 proteins specific for this fraction) (Figure 2A; the Supplementary Material Table S2).

We looked for biological functions associated with proteins characteristic for exosomes present in fraction F7 (the Supplementary Material Table S3). Biological processes associated with the most numerous groups of such proteins included signal transduction, regulation of metabolic processes, regulation of development and differentiation, and transport (including 25 proteins associated with GO term “vesicle-mediated transport”). Moreover, these processes included regulation of gene expression, receptor signaling pathways, regulation of the immune system, and regulation of cell death (Figure 2B). Exosomes are known mediators in the regulation of immune functions. Therefore, it is important to note that about 30 proteins characteristic for fraction F7 were associated with different aspects of immune and inflammatory response (namely ADIPOQ, ARHGAP9, C1RL, CD5L, CD44, CDC5L, CRACR2A, CYP26B1, DCD, FASN, FCN2, FZD8, GPR84, IL1RAP, IRS2, LGALS3BP, MAP2K7, MASP1, MASP2, MBL2, MYH9, NKX3-1, PIGR, PRKDC, PSMA1, S100A8, S100A9, SSC5D, TEC, THBS1, and TRIM25). Furthermore, a large fraction of proteins detected in fraction F7 (45 proteins) were associated with “extracellular exosome” localization (GO:0070062) (Figure 2B).

Finally, we looked for differences in biological functions associated with proteins specific for fraction F7 and proteins specific for fraction F9 (the major serum proteins excluded) (Figure 2C and the Supplementary Material Table S4). Clusters of biological processes (BP) overrepresented in fraction F7 included response to hormone and cytokine stimulus, regulation of membrane potential, regulation of transferases and (apoptotic) endopeptidases and leukocyte aggregation, which corresponded to functions that could be expected for exosomes participating in communication between immune cells. On the other hand, processes involved in ribosome assembly were associated with proteins specific for fraction F9. Furthermore, molecular functions (MF) overrepresented in fraction F7 included binding to regulatory DNA sequences, binding to cytoskeleton proteins and protein kinases, and signaling receptor activity. Moreover, cellular compartments (CC) overrepresented in case of proteins specific for fraction F9 included Golgi and vacuolar membrane, and adherence junctions (Figure 1C).

## 4. Discussion

Serum and plasma represent a very specific type of protein mixture with an extremely wide range of concentrations from 5 × 10^−2^ g per mL (albumin) to 1 x 10^−9^ g per mL (e.g., PSA), where 30 of the most abundant proteins comprise >99 percent of its total protein mass. Therefore, the detection of a specific low abundance serum protein by an untargeted shotgun approach always represent a problematic issue [29]. Moreover, due to unavoidable “contamination” of EVs purified from this specimen with abundant serum components, identification of EV-specific proteins also remains a technical challenge. Several reports indicated that exosomes isolated from plasma using the size-exclusion chromatography (SEC) standalone could be successfully used for testing their biological activity [21,22]. However, proteomic studies that addressed SEC-purified serum/plasma EVs reported rather few exosome-specific proteins that could be identified by untargeted LC–MS/MS approaches, mostly due to co-purification of the abundant serum proteins. De Menezes-Neto and coworkers reported 330 proteins in EVs isolated from 1 mL of plasma using the SEC standalone, yet this number of proteins resulted from the combination of proteins detected in different SEC fraction from 3 donors, while the average number of identified proteins in analyzed fraction was usually below 100 (including the major plasma proteins) [17]. Similarly, Karimi and coworkers reported 88 proteins, mostly plasma proteins, in the EV fraction isolated from 1 mL of plasma by the SEC standalone [25]. Therefore, the complementation of the SEC with other separation methods was proposed aimed to increase the number of proteins identified in EVs isolated from human plasma or serum. Loose and coworkers were first to propose a combination of SEC with density gradient centrifugation and ultracentrifugation, which allowed the detection of 66 proteins in material separated by SDS-PAGE (half of them being abundant plasma proteins) [38]. Kalra and coworkers compared three different methods for plasma exosome purification (ultracentrifugation, immune-affinity pull-down, and density gradient centrifugation) and concluded that a density gradient centrifugation using the OptiPrepTM was the most effective. In the EV fraction isolated from 25 mL of plasma by this approach, 184 proteins were identified (including about 30 plasma proteins and 20 keratins) [39]. More recently, Karimi and coworkers used a combination of SEC with ultracentrifugation and density cushion to purify human plasma exosomes, which allowed them to identify by LC–MS/MS about 1200 proteins (including about 200 proteins that should be considered as plasma proteins). In fact, it was the first report showing identification by an untargeted shotgun LC–MS/MS approach of “classical exosome markers” (CD9, CD63, CD81, and other tetraspanins) in EVs isolated from human plasma. However, the proposed approach was rather laborious and required a large amount of material (up to 80 mL of pooled plasma samples) [25]. Therefore, one should conclude that a reliable approach for untargeted identification of exosome proteins by shotgun LC–MS/MS that would be feasible in a routine work with small volumes of serum or plasma (e.g., ≤1 mL) is not available at present.

We have previously reported a proteomics pipeline optimized for identification of exosome proteins purified by the SEC from the cell culture media [30], which allowed to identify above 1200 proteins in EVs released to media by head and neck cancer cell line, including typical exosome markers (CD9, CD63, CD81, ALIX, TSG101, etc.) and excluding co-purified serum proteins from fetal bovine serum complementing culture media [40]. Here we aimed to implement this proteomics protocol for identification of proteins specific for exosomes isolated by the SEC standalone from a small volume of human serum (i.e., ≤1 mL). However, the implemented protocol performed less effectively in the case of this biospecimen. We found that the exosome-containing SEC fraction (i.e., fraction F7), though depleted of the majority of lipoproteins and “soluble” serum components, still contained a large number of apolipoproteins and other major serum proteins. Nevertheless, after the exclusion of “serum contaminants”, we were able to identify 173 unique proteins characteristic for this exosome-containing fraction. Assuming a low amount of starting material and rather a simple method of EV purification, this approach seemed to outperform the abovementioned protocols that also involved the untargeted shotgun LC–MS/MS and comparable methodology of exosome purification. 

Identification of low abundance proteins present in a very complex biological mixture by untargeted shotgun LC–MS/MS approaches somehow represents a stochastic problem, hence the inconsistency of the resulting protein lists is frequently observed. Among 267 proteins detected here in fraction F7 (after immunoglobulin exclusion), there were 107 “exosome proteins” reported in a “reference” paper of Karimi et al. [25] and 74 “exosome proteins” reported in the work of de Menezes-Neto et al. [17]. However, the majority of such overlapped proteins represented abundant serum components. When 173 proteins considered here as “not serum/exosome characteristic” were analyzed, there were 27 and 9 proteins overlapped with protein lists reported in papers [25] and [17], respectively. There were 8 “not serum” proteins that overlapped among all three list, including CD5 antigen-like (CD5L), IgGFc-binding protein (FCGBP), galectin-3-binding protein (LGALS3BP), proteoglycan 4 (PRG4), calgranulin A (S100A8), scavenger receptor cysteine-rich glycoprotein (SSC5D), as well as possible “experimenter contaminations” - keratin 9 (KRT9) and dermcidin (DCD); see the Supplementary Material Table S2 for details. Moreover, there were 4 “not serum” proteins in fraction F7 listed among the Top-100 EV Proteins by the Vesiclepedia (www.microvesicles.org): clathrin heavy chain 1 (CLTC), fatty acid synthase (FASN), galectin-3-binding protein (LGALS3BP), and myosin-9 (MYH9). On the other hand, however, about 25% of “not serum” proteins detected in the SEC fraction F7 was associated with “extracellular exosome” localization according to the gene ontology classification (GO:0070062). Moreover, about 20% of “not serum” present in this fraction was associated with different aspects of immune-related function (based on the GO classification), which represent a key role for exosomes released by immune cells present in blood [41]. It is also noteworthy that Western blot (and immunogold-EM) revealed enrichment of CD63 and CD81 but not of CD9 and TSG101 in the exosome-containing fraction. A similar observation was reported regarding the lack of TSG101 enrichment in exosome-containing fraction purified from plasma by a combination of SES and density cushion [25]. Therefore, one should be aware that not all proteins considered as “exosome markers” are equally good markers of EVs present in human serum or plasma.

## 5. Conclusions

We concluded that proteins present in the exosome-containing fraction purified by the micro-SEC standalone from a small amount of human serum and identified by untargeted shotgun LC–MS/MS approach represent a bona fide content of these vesicles. However, this relatively simple approach did not warrant a complete coverage of exosome proteome, mainly due to the presence of co-purified high abundance serum proteins. Therefore, to increase the completeness of exosome proteome coverage, a higher degree of sample purity is necessary that could be achieved by a combination of multiple isolation approaches. Available protocols include the combination of SEC, ultracentrifugation and density gradient centrifugation that usually require a large amount of biological material (tens of mL of plasma/serum), which is an unfavorable condition in many clinic-oriented studies. Therefore alternative approaches that require a small amount of material (<1 mL of serum/plasma) should be offered for proteomics (and metabolomics) studies, which would be feasible for retrospective studies based on material stored in biobanks. More recently a protocol combining the micro-SEC and immune-affinity chromatography was used to purify tumor-derived exosomes from a small amount of plasma of melanoma patients [16]. Hence, it is noteworthy that application of proteomics protocols described in the current work allowed detection by LC–MS/MS of more than 500 proteins characteristic for melanoma-derived exosomes including typical exosome markers (CD9, CD63, CD81, etc.) with a very low number of abundant plasma proteins (Pietrowska et al.; unpublished data). Nevertheless, the protocol proposed in the current paper could be recommended for pilot exploratory studies when a small amount of a serum/plasma specimen is available. However, analysis of resulting data should include removal of serum proteins (e.g., based on a list of proteins identified in the unfractionated specimen) that inevitably co-purify with exosome preparation.

## Supplementary Materials

The following are available online at https://www.mdpi.com/2227-7382/7/2/18/s1, Figure S1: Characteristics of vesicles purified from serum of four different donors, Table S1: Proteins and their peptides identified in either F7 and F9 SEC fractions, Table S2: Proteins detected in fractions F7 and F9, Table S3: GO terms associated with proteins identified in fractions F7 (after exclusion of serum proteins), Table S4: Comparison of GO terms associated with proteins specific for fraction F7 and F9.
