# Supplementary material for: Proteome Profiling of Exosomes Purified from a Small Amount of Human Serum: The Problem of Co-Purified Serum Components
Source: Proteomes. 2019 Apr 28;7(2):18. doi: 10.3390/proteomes7020018 (PMC6630217; doi:10.3390/proteomes7020018)
Supplement: Supplementary file 1 [file proteomes-07-00018-s001.zip › Supplementary Material/Supplementary Figure S1.pdf]

Proteome profiling of exosomes purified from a small amount of human serum: the problem of co-purified serum components.

Mateusz Smolarz et al.

Supplementary Material Figure S1.

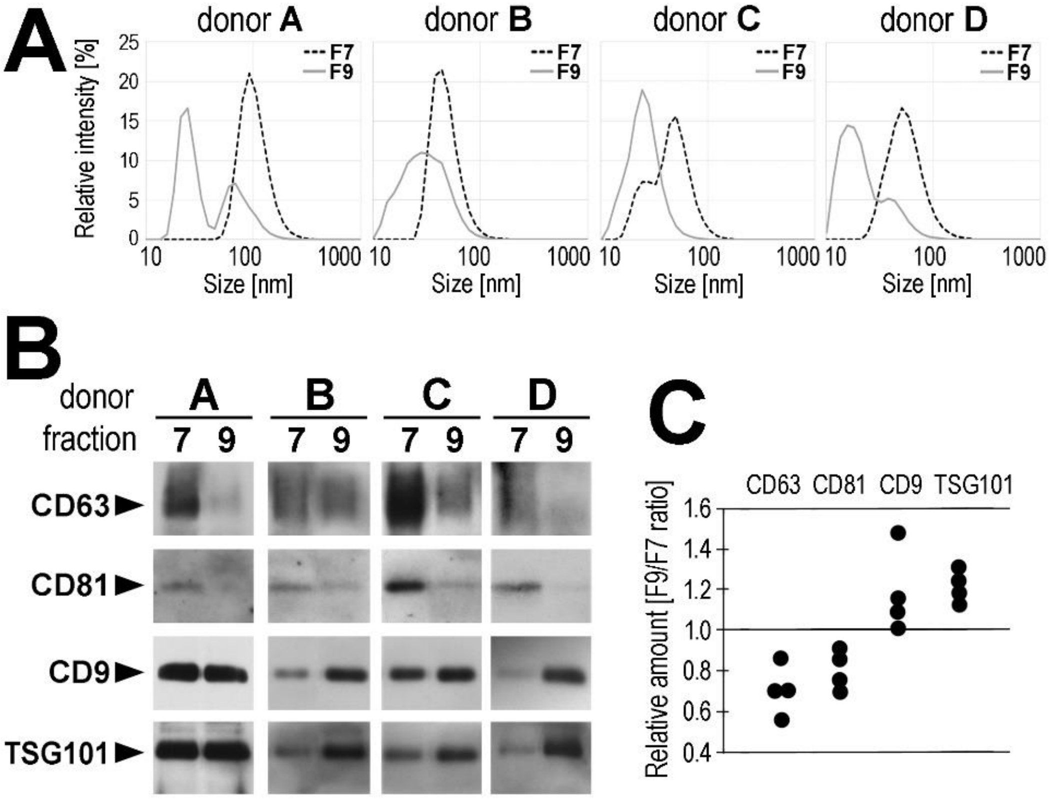

Supplementary Figure S1. Characteristics of vesicles purified from serum of four different donors. **Panel A** – Size of vesicles measured by DLS in fractions F7 and F9. **Panel B** – Level of selected exosome proteins analyzed by Western-blot in fractions F7 and F9; the same amount of total proteins (0.2 µg) was used for both fraction. **Panel C** – Relative amount of selected proteins in both fractions (the F9/F7 ratio); each donor is represented by a dot.
